# Risk Factors and Outcomes of Treatment Delays in Lyme Disease: A Population-Based Retrospective Cohort Study

**DOI:** 10.3389/fmed.2020.560018

**Published:** 2020-11-26

**Authors:** Annemarie G. Hirsch, Melissa N. Poulsen, Cara Nordberg, Katherine A. Moon, Alison W. Rebman, John N. Aucott, Christopher D. Heaney, Brian S. Schwartz

**Affiliations:** ^1^Department of Population Health Sciences, Geisinger, Danville, PA, United States; ^2^Department of Environmental Health and Engineering, Johns Hopkins University Bloomberg School of Public Health, Baltimore, MD, United States; ^3^Division of Rheumatology, Department of Medicine, Johns Hopkins University School of Medicine, Baltimore, MD, United States; ^4^Department of Epidemiology, Johns Hopkins University Bloomberg School of Public Health, Baltimore, MD, United States; ^5^Department of International Health, Johns Hopkins University Bloomberg School of Public Health, Baltimore, MD, United States

**Keywords:** Lyme disease, treatment delays, post-treatment Lyme disease syndrome, time-to-treatment, disparities

## Abstract

**Background:** Longer time between symptom onset and treatment of Lyme disease has been associated with poor outcomes. Reducing time-to-treatment requires knowledge of risks for treatment delays. We conducted a population-based study to evaluate factors associated with delayed treatment of Lyme disease and the relation between delayed treatment and post-treatment Lyme disease syndrome (PTLDS).

**Methods:** We mailed questionnaires to 5,314 individuals with a Lyme disease diagnosis or blood test followed by an antibiotic order in the medical record of a Pennsylvania health system from 2015 to 2017. Analyses were confined to 778 respondents who reported that they were treated for Lyme disease within the past 5 years and reported a rash and/or a positive blood test for Lyme disease. Time-to-treatment was calculated as the sum of two windows before and after seeking care for Lyme disease symptoms: time to first medical contact and time under care. We used logistic regression to evaluate factors associated with delayed time-to-treatment in each time window (>14 days vs. ≤14 days) and the association between total time-to-treatment (>30 days vs. ≤30 days) and PTLDS. We used inverse probability weighting to calculate estimates for the study's source population (5,314 individuals sent questionnaires).

**Results:** In the source population, 25% had time to first contact >14 days, 21% had time under care >14 days, and 31% had a total time-to-treatment >30 days. Being uninsured and attributing initial symptoms to something other than Lyme disease were positively associated with delayed time to first medical contact, while seeking care at an urgent care or emergency setting (vs. primary care) was negatively associated. Diagnoses between November and April, and the absence of rash were positively associated with delays. Individuals whose treatment was delayed, defined as time-to treatment >30 days had 2.26 (95% confidence interval: 1.25, 4.05) times the odds of PTLDS as those who were treated within 30 days of symptom onset.

**Conclusions:** In a population-based study in Pennsylvania, one-third of Lyme disease patients reported delayed treatment, which was associated with PTLDS. To improve Lyme disease outcomes, prevention efforts should aim to reduce the time before and after seeking care.

## Introduction

Lyme disease is on the rise in the United States, with almost 30,000 confirmed and over 13,000 probable cases in 2017 (1). Delayed treatment can lead to disseminated infection and serious complications (2, 3). Longer time between symptom onset and treatment (time-to-treatment) has been associated with poor Lyme disease outcomes (4–7). Post-treatment Lyme disease syndrome (PTLDS) is characterized by persistent or recurrent symptoms, lasting 6 months or more of fatigue, musculoskeletal pain, and cognitive complaints leading to decline in physical and social functioning (3, 8). The role of time-to-treatment in PTLDS remains unknown. Timely treatment may be important in preventing PTLDS and other long-term consequences of Lyme disease. Strategies to ensure timely treatment require a better understanding of the risk factors for treatment delays.

Of the few studies of time-to-treatment in Lyme disease, most have been confined to individuals with Lyme neuroborreliosis, a neurological manifestation of disseminated Lyme disease that occurs in about 12% of Lyme disease cases (4–7, 9). These studies have reported that longer time-to-treatment is associated with poor outcomes, including persistent Lyme disease symptoms and poor quality-of-life. No studies have evaluated the role of time-to-treatment in PTLDS, a condition that occurs in an estimated 10 to 20% of Lyme disease cases (10). PTLDS is a well-defined condition that is distinct from chronic Lyme disease, a non-specific term that has been used to describe illness in individuals with Lyme disease and around which there is ongoing debate (11). The biological basis for PTLDS is not well-understood, and no evidence-based treatment has been identified (8). Thus, exploring options for prevention is critical.

Evidence-based strategies for reducing time-to-treatment of Lyme disease are lacking, in part due to limited understanding of related risk factors. Prior studies have generally measured time-to-treatment of Lyme disease as a single time period (5–7). However, the General Model of Total Patient Delay, a widely used model that describes stages of treatment delay, differentiates the time before and after a patient sees a medical professional (12). The time between symptom onset and seeing a medical professional (hereafter, “time to first medical contact”) and the time while under the care of a medical professional until receiving treatment (hereafter, “time under care”) involve different actors and occur in different settings. Thus, these stages may have distinct risk factors that require different approaches for promoting timely treatment.

We conducted a retrospective cohort study of time-to-treatment among a general population sample of individuals treated for Lyme disease at Geisinger, a health system in Pennsylvania, the state with the most confirmed Lyme disease cases in the United States (13). Using self-administered questionnaire data, we characterized respondents' experiences with Lyme disease symptoms, care-seeking, diagnosis, and treatment; measured risk factors for delays in time to first medical contact and time under care; and examined associations between time-to-treatment and PTLDS.

## Methods

### Study Population

Participants were identified through the Geisinger electronic health record (EHR). Geisinger serves patients across 45 Pennsylvania counties. The primary care population represents the age and sex distribution of the region's population (14). We mailed questionnaires to 5,314 adult patients who met previously described EHR-based criteria for Lyme disease between 2015 and 2017 (15). Briefly, individuals had to have a Lyme disease diagnostic code (*International Classification of Diseases*, 9th Revision, code 088.81) or both a *Current Procedural Terminology* code for a Lyme disease serologic test (enzyme immunoassay or Western blot) and an antibiotic order appropriate for Lyme disease, regardless of length of treatment, within 30 days after the sample draw. Appropriate treatment was defined by the Infectious Disease Society of America's (IDSA) recommended first or second line antibiotics (3) and three antibiotics either closely related to recommended treatments or that were historical treatments (15). We excluded antibiotic orders if the diagnosis codes linked to the medication orders were for respiratory disease, since these are common diagnoses treated with the same antibiotics as Lyme disease. A $1 bill was included with the questionnaire. Non-respondents were re-sent a questionnaire 6 weeks after the original mailing. Geisinger's Institutional Review Board approved the study.

### Questionnaire Development

We developed a questionnaire to measure time-to-treatment for Lyme disease and potential related factors and outcomes, informed by interviews with Lyme disease patients and physicians (16). Based on findings from this formative work, a panel of experts specializing in epidemiology, survey research, infectious disease, and rheumatology developed the questionnaire. Questions were derived from existing instruments or created *de novo* based on scientific literature.

### Time-to-Treatment

Time-to-treatment was measured (in days) as the sum of two time windows: time to first medical contact and time under care. Time to first medical contact was based on response to the question, “About how long did you wait after your first symptom of Lyme disease before contacting a medical professional?” Time under care was based on response to the question, “How long was it from your first contact with a medical provider to when you were treated for Lyme disease?”

### PTLDS

PTLDS was defined based on criteria developed by Aucott et al. (8), consistent with guidelines from the IDSA (3). Participants were classified as having PTLDS if they had received antibiotic treatment for Lyme disease and reported persistent symptoms and functional deficit. Respondents were classified as having persistent symptoms if they reported that one of the following symptoms had not changed, had worsened, or had newly occurred in the 6 months after completing antibiotic treatment for Lyme disease: fatigue, muscle pain, joint pain, memory changes, difficulty finding words, or difficulty focusing. Functional deficit was defined as a standardized T score <45 of the mean of the following subscales from the 36-Item Short Form Health Survey: role limitations due to physical health, energy/fatigue, emotional well-being, or role limitations due to emotional health (10, 17). Consistent with IDSA guidelines, a participant could not be classified as having PTLDS if they reported a prior diagnosis of fibromyalgia or chronic fatigue syndrome (CFS) (3).

### Lyme Disease Symptoms, Care-Seeking, Diagnosis, and Treatment

The questionnaire captured the respondents' experiences related to Lyme disease symptoms, care-seeking, diagnosis, and treatment. Items related to Lyme disease symptoms included whether the respondent observed a tick bite or a rash, whether the rash was a bull's-eye rash, the constancy of symptoms, and to what condition respondents initially attributed their Lyme disease symptoms. Items related to care-seeking included specialty of the first medical professional contacted for Lyme disease symptoms, reason for contacting the medical professional, and barriers to contacting a medical professional. The questionnaire also assessed diagnosis received at the first medical visit, whether an antibiotic was prescribed, number of medical professionals seen before receiving a diagnosis of Lyme disease, and blood testing results.

### Coping

Coping was assessed using the John Henry Active Coping Scale, a 12-item scale that assesses a personality pre-disposition to cope with psychosocial stressors (18). Items were summed for a total score ranging from 12 to 60, then dichotomized at the median to categorize respondents into low and high active coping groups.

### Clinical and Demographic Characteristics

Through the questionnaire, we assessed history of a diagnosis prior to Lyme disease of cancer, fibromyalgia, CFS, rheumatoid arthritis, migraine, depression, and anxiety, as well as marital status, income, education, occupation, and insurance status at the time of Lyme disease diagnosis. Age and sex were obtained from the EHR.

### Statistical Analysis

The goals of the analysis were to describe time-to-treatment in a population-based sample of individuals treated for Lyme disease, to identify risk factors for the two time-to-treatment delay windows, and to evaluate associations between time-to-treatment and PTLDS. Analyses were confined to respondents who self-reported a Lyme disease diagnosis within the past 5 years, completed questions related to time-to-treatment and rash, whose Lyme disease was confirmed based on self-report of a rash and/or a positive blood test for Lyme disease, who reported being prescribed antibiotics, and for whom time-to-treatment was plausible (i.e., less than their age) (*n* = 778). We used inverse probability weighting based on EHR-based characteristics available on responders and non-responders to calculate estimates for the source population of the study (the 5,314 individuals sent questionnaires).

We conducted chi-square tests to evaluate the proportion of individuals with delays in time to first medical contact and time under care by the following variables: season of diagnosis (November–April, May–October); presence of rash (yes, no); symptom attribution (Lyme disease, other condition); first medical professional contacted [primary care, urgent care, emergency department, other (e.g., inpatient or specialist)]; self-reported diagnosis of cancer, fibromyalgia, CFS, rheumatoid arthritis, migraine, depression, and anxiety (yes, no); age (18–39, 40–49, 50–59, 60–69, ≥ 70 years); sex (male, female); insurance at time of diagnosis (private insurance, Medicaid, no health insurance, Medicare); education (less than high school, high school graduate, some college, associate degree, bachelor's degree, graduate degree); and marital status (never married, separated/divorced/widowed, married or living with a partner). For each time window a delay was described as a period lasting more than 14 days. Next, we used logistic regression to evaluate factors associated with treatment delays, separately for time to first medical contact and time under care (>14 days vs. ≤ 14 days). All models controlled for age (continuous), sex, insurance status, rash, and season of diagnosis. Age was tested for linearity. Additional variables that demonstrated a bivariate association with the treatment delay were added to models individually. The final models retained variables that remained associated with the treatment delay using a threshold of *p* < 0.05. We used robust standard errors, calculated using the Huber–White sandwich estimator. Model diagnostics were performed to confirm the validity of multivariable models. Hosmer–Lemeshow tests and F-tests were used to assess goodness-of-fit, while scatterplots of standardized residual vs. predicted probability of outcome were used to look for influential observations (Supplementary Material).

We used logistic regression to evaluate the association between time-to-treatment (sum of time to first medical contact and time under care, >30 days vs. ≤ 30 days) and PTLDS (yes vs. no). The base model included age-centered, age-centered squared, sex, insurance, and time-to-treatment. We evaluated the following variables for confounding: self-reported prior diagnosis of cancer, migraine, rheumatoid arthritis, depression, or anxiety; education; occupation; marital status; and coping score (< median vs. ≥ median). Variables were retained if adding the variable to the model changed the estimate of the association between time-to-treatment and PTLDS by at least 10%. We evaluated whether depression, anxiety, rash, and coping modified the association between time-to-treatment and PTLDS by adding cross-product terms (separately for each interaction) to the model. The same model diagnostics described above were performed. Analyses were conducted using Stata 14.1 (19).

## Results

### Demographic and Clinical Characteristics

**Figure 1:**
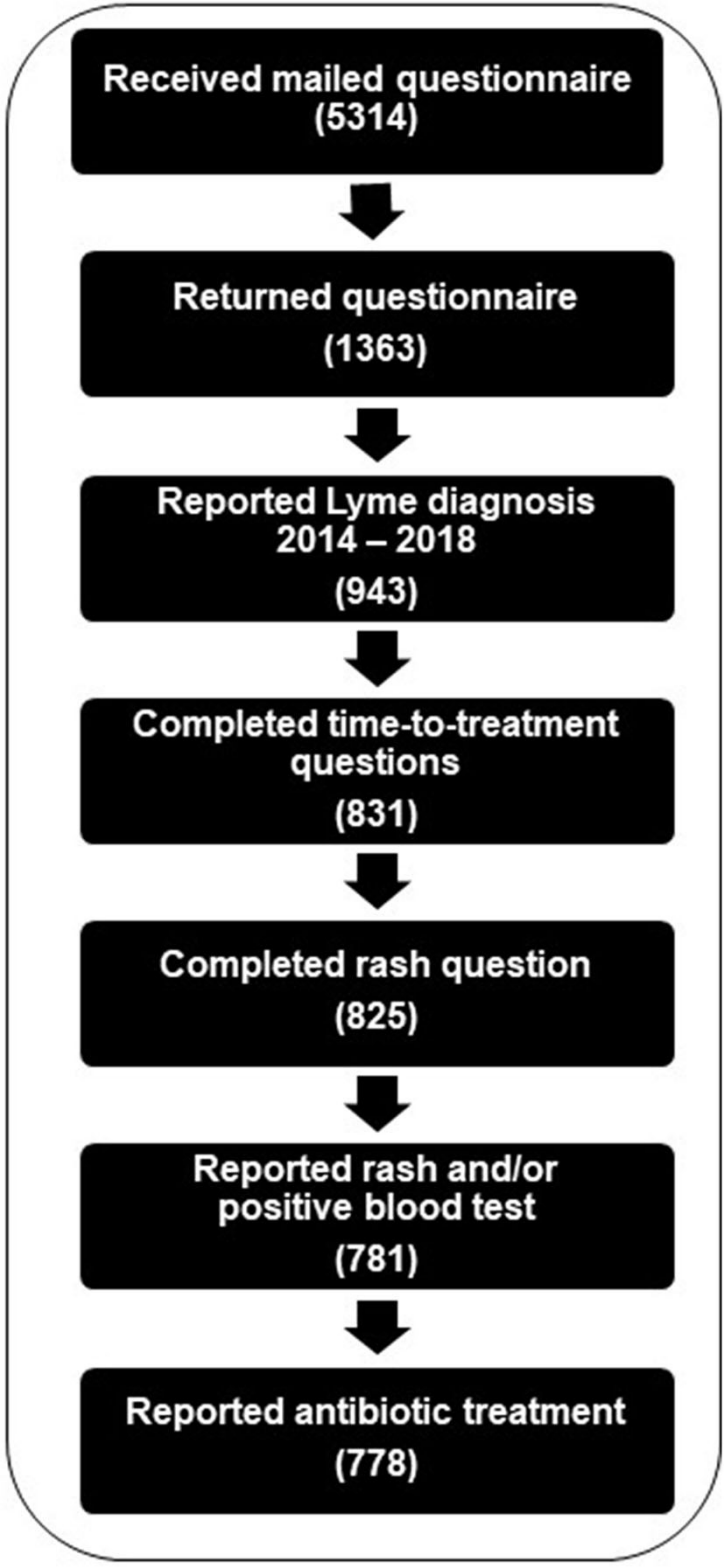
Creation of analytic dataset of respondents to the Lyme disease time-to-treatment questionnaire, with inclusion based on responses to questionnaire items regarding date of Lyme disease diagnosis, completion of time-to-treatment and rash questions with plausible response, report of rash and/or blood test, and report of antibiotic treatment.

Of the 5,314 individuals who received a questionnaire, 1,364 returned a completed questionnaire, of whom 778 met the inclusion criteria for the analysis (Figure 1). Because weighted analysis accounts for potential participation bias, only weighted results are described in the text; both unweighted and weighted results are presented in tables. A little less than half of the study population was female and the mean age was 51 years (Table 1). At the time of Lyme disease diagnosis, 78% of the study population had private health insurance, 3% were not insured, and the remaining were insured with Medicare or Medicaid. An estimated 11.5% of the study population met the criteria for PTLDS.

**Table 1:** Characteristics of study population, with unweighted and weighted percentages.

	**Frequencies unless otherwise noted**		
	***N***	**Unweighted %**	**Weighted %**^**a**^
Total respondents	778	100	n/a
Age in years, mean		57	51
18–39	131	17	29
40–49	107	14	16
50–59	152	20	19
60–69	236	30	22
≥70	152	20	13
Female	401	52	48
Education			
Less than high school	53	7	9
High school graduate	233	30	28
Some college	141	18	20
Associate degree	84	11	11
Bachelor's degree	139	18	18
Graduate degree	128	16	15
Marital status			
Never married	83	11	16
Separated, divorced, or widowed	107	14	13
Married or living with a partner	588	76	71
Self-reported health insurance status^b^			
Medicaid (with or without Medicare)	52	7	10
Medicare only	91	12	8
No health insurance	22	3	3
Private insurance	613	79	78
Self-reported diagnoses prior to Lyme disease^c^			
Cancer	74	10	8
Fibromyalgia	26	3	3
Chronic fatigue syndrome	18	2	2
Rheumatoid arthritis	54	7	6
Migraine	88	11	11
Depression	137	18	17
Anxiety	142	18	19
PTLDS^d^			
Yes	75	10	12
No	693	89	87
Missing	10	1	1

*PTLDS, post-treatment Lyme disease syndrome*.

a *Weighted by participation rates*.

b *Self-reported insurance coverage at time of Lyme diagnosis*.

c *Self-reported diagnosis by a doctor that occurred prior to Lyme disease*.

d *PTLDS based on self-reported new or persistent symptoms and functional impairment after treatment, excluding those with prior diagnosis of chronic fatigue syndrome or fibromyalgia*.

### Time-to-Treatment

Median time-to-treatment was 13 days (Table 2). An estimated 31% of study population had time-to-treatment >30 days. One-quarter reported time-to-first contact with a medical professional >14 days and 21% reported time under care >14 days. Among those with total time-to-treatment >30 days, the average ratio of time to first medical contact to time under care was 1:1, with an equal contribution of time from both delay windows.

**Table 2:** Symptom, care-seeking, diagnostic, and treatment experiences for Lyme disease among survey respondents (*n* = 778), with unweighted and weighted proportions.

	**Frequencies unless otherwise noted**		
	***N***	**Unweighted %**	**Weighted %**
**Time to treatment for Lyme disease**			
Days from first symptoms to contacting a medical professional, median (range)	7 (0, 5,479)		
0–14 days	601	77	75
>14 days	177	23	25
Days from healthcare contact to treatment, median (range)	2 (0, 13,880)		
0–14 days	634	81	79
>14 days	144	19	21
Total days from first symptoms to treatment, median (range)	13 (0, 13,890)		
0–4 days	203	26	24
>4–14 days	215	28	27
>14–30 days	142	18	18
>30 days−6 months	149	19	21
>6 months	69	9	10
**Experiences with Lyme disease symptoms**			
Observed a tick bite	214	28	28
Reported rash^**a**^			
Experienced a typical bull's-eye rash	372	48	46
Experienced a rash (not bull's-eye)	163	21	20
No rash	239	31	33
Constancy of symptoms^**a**^			
Symptoms were constant	242	31	31
Symptoms would come and go	92	12	11
Some constant, some would come and go	375	48	51
Attributed first symptoms to Lyme disease	167	21	21
Misattributed first symptoms to other conditions^b^			
Flu or virus	251	32	34
Bug bite, allergy, or skin problem	127	16	15
Muscle or joint strain/injury	89	11	12
Arthritis or bursitis	80	10	10
Dehydration, overexertion, stress, old age	22	3	3
Other	49	6	8
Did not know	41	5	5
**Experiences seeking medical care for Lyme disease symptoms**			
Did not wait to contact a medical professional	421	54	51
Barriers to contacting a medical professional^b^			
Symptoms perceived to not be serious or were attributed to another cause	321	41	43
Socioeconomic barriers (e.g., cost, transportation, caregiving duties)	41	5	7
Immediate healthcare not accessible (e.g., appointments unavailable, traveling)	21	3	4
Reason for contacting a doctor^b^			
Suspected Lyme disease (e.g., tick bite, bull's-eye rash, previous experience)	95	12	11
New symptoms appeared	152	20	22
Symptoms did not go away	340	44	44
Symptoms got more severe	315	40	43
Symptoms interfered with work or daily tasks	175	22	27
Family or friend said to go	146	19	19
**Experiences with diagnosis and treatment for Lyme disease symptoms**			
First medical professional contacted about symptoms^a^			
Urgent care	190	24	25
Emergency department	85	11	12
Primary care	477	61	61
Other^c^	25	3	3
Diagnosis received at first medical visit^a^			
Lyme disease or suspected Lyme disease^d^	455	58	56
Flu or other viral infection	50	6	6
Skin rash, allergic reaction, shingles	47	6	6
Muscle or joint injury	30	4	5
Cellulitis or other skin infection	23	3	3
Insect bite	22	3	3
Arthritis	5	1	1
Other	36	5	5
None	97	12	13
Number of medical professionals seen for Lyme disease symptoms before receiving a Lyme disease diagnosis^a^			
0–1	423	54	52
2	140	18	19
≥3	91	12	13
Medical care provider who diagnosed respondent's Lyme disease^a^			
Urgent care clinic doctor	154	20	19
Emergency department doctor	72	9	9
Primary care or family doctor	432	56	55
Specialist (e.g., rheumatologist, cardiologist, neurologist, infectious disease doctor)	75	10	10
Lyme specialist	25	3	4
Self-diagnosis or other non-medical diagnosis	10	1	1
Diagnosis season^a^			
May–October	582	75	74
November–April	136	17	18
Blood testing^a^			
First test was positive	501	64	63
First test was negative, second test was positive	102	13	16
Blood tests only negative	47	6	6
Blood never tested	110	14	13
Received antibiotic treatment at first medical visit			
Yes	542	70	68
No	236	30	32
Lyme disease treatment received^a^			
1 oral antibiotic	556	71	70
1 intravenous antibiotic or 2 oral antibiotics	135	17	17
>2 antibiotics	76	10	11

a *Categories do not add to 100% of sample due to missing data*.

b *Categories are not mutually exclusive*.

c *“Other” includes specialists (e.g., dermatologist) and inpatient/hospital*.

d *Respondent indicated there was no diagnosis, but blood testing was ordered*.

### Experiences With Lyme Disease Symptoms, Care-Seeking, Diagnosis, and Treatment

Forty-six percent of the study population reported having a bull's-eye rash, and 20% reported a rash without central clearing. About one-fifth (21%) of the population attributed their initial symptoms to Lyme disease; the remaining attributed initial symptoms to flu or a virus (34%); a bug bite, allergy, or skin problem (15%); a muscle or joint strain/injury (12%); bursitis (10%); or a mix of other conditions (Table 2). Nearly half of the study population reported they did not immediately contact a medical professional largely because initial symptoms were not perceived to be serious.

The majority of the study population reported initially seeking care from a primary care provider (61%). Urgent care was the first contact for an estimated 25% of the population. An estimated 56% received a diagnosis of Lyme disease at their initial medical visit (Table 2), though 68% of the study population reported receiving antibiotic treatment at their first visit. Most diagnoses (74%) occurred between May and October.

### Factors Associated With Delayed Time to First Medical Contact

In bivariate analyses, factors associated with delayed time to first contact with a medical professional (>14 days) included younger age, no rash, Lyme disease diagnosis between November and April, misattribution of symptoms, being uninsured, first medical contact in an urgent care or emergency department setting, and self-reported diagnosis of cancer. In a model adjusted for age, sex, presence of rash, and diagnosis season, the odds of delayed time to first medical contact among those who reported being uninsured was 3.49 [95% confidence interval (CI): 1.19, 10.21] times the odds of those with private insurance. The odds of delayed time to first medical contact among those who initially attributed their symptoms to something other than Lyme disease was 3.51 (95% CI: 1.79, 6.89) times the odds of those who initially attributed symptoms to Lyme disease (Table 3). Odds of delay among individuals who initially sought care in an urgent care or emergency department setting were 0.33 (95% CI: 0.17, 0.64) and 0.37 (0.17, 0.81), respectively, times the odds of those who sought care from a primary care provider. The odds of delay among who reported a rash was 0.44 (95% CI: 0.27, 0.71) times the odds among those without rash.

**Table 3:** Logistic regression analysis of factors related to delays in contacting a medical professional^a^ for Lyme disease.

	**Study sample (*n* = 717^**b**^) unweighted**	**Source population weighted^**c**^**
**Respondent characteristic**	**Odds ratio (95% CI)**	**Odds ratio (95% CI)**
Age	0.98 (0.97, 1.00)	0.97 (0.96, 0.99)
Sex, female	0.73 (0.50, 1.08)	0.70 (0.44, 1.10)
Insurance^d^		
Privately insured	Ref	Ref
Medicaid only or with Medicare	1.03 (0.51, 2.07)	1.26 (0.61, 2.62)
No health insurance	3.09 (1.21, 7.86)	3.49 (1.19, 10.21)
Medicare only	1.50 (0.77, 2.92)	1.84 (0.92, 3.69)
Presence of rash	0.39 (0.26, 0.58)	0.44 (0.27, 0.71)
Diagnosis season		
May–October	Ref	Ref
November–April	2.20 (1.42, 3.41)	2.60 (1.60, 4.21)
Attributed first symptoms to Lyme disease		
Yes	Ref	Ref
No	2.93 (1.67, 5.14)	3.51 (1.79, 6.89)
First medical provider contacted about Lyme disease symptoms		
Primary care/family doctor	Ref	Ref
Urgent care clinic	0.38 (0.22, 0.66)	0.33 (0.17, 0.64)
Emergency department	0.49 (0.27, 0.89)	0.37 (0.17, 0.81)
Other^e^	1.48 (0.61, 3.60)	1.23 (0.44, 3.44)

a *Delay characterized as >14 days (vs. ≤ 14 days) from first symptoms of Lyme disease to contacting a medical professional, as reported by respondents*.

b *Data on rash, diagnosis season, and first medical provider contacted about Lyme disease symptoms missing for 61 respondents*.

c *Weighted by participation rates*.

d *Self-reported insurance coverage at time of Lyme diagnosis*.

e *“Other” includes specialists (e.g., dermatologist) and inpatient/hospital*.

### Factors Associated With Time Under Care

In bivariate analyses, factors associated with delayed treatment while under care of a medical professional (>14 days) included younger age; never married; unable to work/disabled; no rash; Lyme disease diagnosis between November and April; first medical contact in an emergency department or “other” setting; and self-reported diagnosis of fibromyalgia, CFS, or migraine prior to Lyme disease. In models adjusted for age, sex, and insurance status, rash was associated with nearly half the odds of delay under care (Table 4). The odds of the delay among those diagnosed between November and April was 2.36 (95% CI: 1.37, 4.07) times the odds of those diagnosed at other times of the year. The odds of delay among those with a diagnosis of chronic fatigue syndrome was 5.02 (95% CI: 1.79, 14.12) times the odds among those without a diagnosis.

**Table 4:** Logistic regression analysis of factors related to delays between healthcare contact and treatment^a^ for Lyme disease.

	**Study sample (*n* = 718^**b**^) unweighted**	**Source population weighted^**c**^**
**Respondent characteristic**	**Odds Ratio (95% CI)**	**Odds Ratio (95% CI)**
Age	0.98 (0.97, 1.00)	0.98 (0.96, 1.00)
Sex, female	0.96 (0.64, 1.43)	1.06 (0.66, 1.71)
Insurance^d^		
Privately insured	Ref	Ref
Medicaid only or with Medicare	1.43 (0.72, 2.84)	1.09 (0.48, 2.50)
No health insurance	1.13 (0.41, 3.18)	1.13 (0.40, 3.21)
Medicare only	0.51 (0.22, 1.17)	0.75 (0.25, 2.28)
Rash accompanied Lyme disease	0.52 (0.34, 0.78)	0.56 (0.34, 0.91)
Diagnosis season		
May–October	Ref	Ref
November–April	2.07 (1.32, 3.25)	2.36 (1.37, 4.07)
Chronic fatigue syndrome^e^	5.03 (1.90, 13.29)	5.02 (1.79, 14.12)

a *Delay characterized as >14 days (vs. ≤ 14 days) from first contact with a medical provider to treatment for Lyme disease, as reported by respondents*.

b *Data on rash and diagnosis season missing for 60 respondents*.

c *Weighted by participation rates*.

d *Self-reported insurance coverage at time of Lyme diagnosis*.

e *Self-reported diagnosis (yes vs. no) by a doctor that occurred prior to Lyme disease*.

### Time-to-Treatment and PTLDS

The odds of PTLDS among those with time-to-treatment >30 days was 2.26 (95% CI: 1.25, 4.05) times the odds of those treated within 30 days, adjusting for age (centered and centered-squared), sex, and insurance status. Depression, anxiety, presence of rash, and coping did not modify the association between time-to-treatment and PTLDS.

## Discussion

In this first population-based study of time-to-treatment of Lyme disease, we characterized experiences with Lyme disease symptoms, care-seeking, diagnosis, and treatment among individuals in Pennsylvania, a state highly endemic to Lyme disease; identified common and unique factors associated with delays before and after contacting a medical professional; and evaluated long-term consequences of delayed treatment. In a novel finding, we observed that time-to-treatment was associated with PTLDS, demonstrating the potential long-term consequences of delayed treatment. Several factors—including insurance status, the presence of a rash, diagnosis season, attribution of initial symptoms to Lyme disease, the first medical provider contacted about the symptoms, and a diagnosis of chronic fatigue syndrome prior to Lyme disease—were related to treatment delays. These findings have important implications for strategies to reduce time-to-treatment in Lyme disease and the potential of these efforts to improve long-term outcomes.

We found that delayed treatment was associated with higher risk of PTLDS. Although our study is the first to evaluate time-to-treatment in relation to PTLDS, the findings are consistent with prior studies that examined persistent symptoms. Rebman et al. (11) found that in a sample of individuals with PTLDS, 45% reported time-to-treatment >30 days. Negative consequences of delayed treatment for Lyme disease have been previously reported, with longer time-to-treatment associated with persistent symptoms, poor quality-of-life, and Lyme neuroborreliosis, but none, to our knowledge, have demonstrated an association with PTLDS specifically (4–7, 20). The benefit of shorter time-to-treatment has been attributed to the prevention of pathogen dissemination, resulting from earlier eradication of the *Borrelia burgdorferi* bacterium (5). Alternative hypotheses for the benefit of early antibiotic treatment include early interruption of the immune response, which may prevent secondary autoimmune reactions (5). While the pathogenesis of PTLDS remains unknown, an autoimmune response is one of the hypothesized causes (8).

Averting treatment delays in Lyme disease may be a key strategy for preventing PTLDS and other serious complications. Prior studies of Lyme disease have defined treatment delay as the time between symptom onset and treatment, with definitions of delay ranging from >30 days to >6 weeks (4, 5, 7, 11). We found that time-to-treatment >30 days has potentially important implications for Lyme disease outcomes, as this delay was associated with more than twice the odds of PTLDS. Of concern, 31% of our study population reported time-to-treatment exceeding 30 days. Other studies have also reported a large proportion of individuals with time-to-treatment longer than 30 days (7, 11). Thus, there remains a substantial delay in Lyme disease care that, if closed, could improve Lyme disease outcomes.

We found that the two time windows comprising time-to-treatment (both before and after contacting a medical professional) contributed equally to Lyme disease treatment delays. One prior study used similar time-to-treatment windows to evaluate individuals with Lyme neuroborreliosis, observing even longer delays than in our study, with a median time from symptom onset to first hospital contact of 20 days and a median time from first hospital contact to treatment of 24 days (4). Thus, there are opportunities to shorten time-to-treatment both before and after an infected individual engages with the healthcare system.

The absence of a rash was a strong factor in delayed treatment for Lyme disease, as it was associated with both delay windows, signifying its importance to both individual and provider behavior. The association of rash with delayed time to medical contact aligns with a prior qualitative study that revealed patients with treatment delays ruled out the possibility of Lyme disease because they did not observe a bull's-eye rash (16). Similar to our findings, past reports indicate that up to 30% of people with Lyme disease do not present with erythema migrans (21) and a subset of these individuals do not present with the characteristic bull's-eye appearance (21). On the healthcare side, misdiagnosis reportedly occurs more commonly among patients with Lyme disease that do not present with erythema migrans (22). This work suggests that efforts to reduce time-to-treatment should include educational campaigns targeting patients and healthcare providers on alternative clinical presentations of Lyme disease and erythema migrans (22).

Delays before and after contacting a medical professional were also more common for Lyme disease diagnosed between November and April compared to other times of the year. A prior study of Lyme neuroborreliosis similarly reported longer time-to-treatment when Lyme disease occurred in winter and early spring (4). This is the time of year when Lyme disease is least commonly contracted (1), thus patients and medical professionals may be less likely to attribute symptoms to Lyme disease in this time period. Though less common in these months, thousands of confirmed cases of Lyme disease are reported from November to April (1). Building awareness among patients and medical providers of the risk of Lyme disease throughout the year in endemic regions provides another opportunity for reducing time-to-treatment.

Uninsured individuals in our study were more likely to delay contacting a medical professional for their symptoms than were individuals with private insurance. This finding aligns with a prior qualitative study of treatment delays in Lyme disease, which highlighted the symptoms that individuals endured while waiting to obtain health insurance, including debilitating joint pain and dangerously high fevers (16). Treatment delays due to lack of insurance occur for a range of conditions, from myocardial infarction (23, 24) to cancer (25), and improving accessibility of health insurance is a critical goal in efforts to provide timely treatment. Considering that the costs of diagnosing and treating acute and uncomplicated Lyme disease are relatively inexpensive (26), diagnostic tests and treatment should be made accessible and affordable for those with and without health insurance.

Most participants reported initially contacting a primary care provider for their Lyme disease symptoms. However, these individuals were at greater risk of delayed treatment than individuals who sought care in an urgent care or emergency department setting. Wait times for primary care appointments can be lengthy, and many primary care clinics do not offer evening or weekend hours (27). In our study, the inability to obtain care outside of work hours or while traveling away from home, and responsibilities such as caregiving duties were noted as barriers to seeking prompt care for Lyme disease symptoms. Urgent care clinics offer an important option for individuals who might otherwise delay contacting a medical professional. Increasing use of urgent care clinics for Lyme disease symptoms may require public health campaigns to inform the general population of the importance of prompt treatment for Lyme disease.

A self-reported diagnosis of CFS prior to Lyme disease increased the odds of delay while under care. Considering the similarity in some symptoms in the two conditions, health care providers may not have initially recognized the onset of Lyme disease symptoms as a new condition, resulting in delayed treatment. Alternatively, CFS may have been later misdiagnosed as Lyme disease, or Lyme disease may have been initially misdiagnosed as CFS (28). Given the small number of individuals in our sample with CFS, these findings should be considered preliminary.

The strengths of this study include a population-based sample from a Lyme endemic state, identification of separate risk factors associated with two time windows of treatment delays that potentially require unique approaches to reducing delays, and evaluation of the association between time-to-treatment and PTLDS using guideline-based criteria that includes persistent symptoms and functional deficit. This study had some limitations. First, we did not require a positive blood test when identifying Lyme disease cases. It is possible that some study respondents did not have Lyme disease, though unlikely given the combination of EHR data—which has demonstrated utility in identifying Lyme disease cases (15)—and self-reported data to identify cases. Confining the study to individuals with a positive blood test would have excluded individuals who were promptly treated with antibiotics or tested before antibodies developed, resulting in an overestimation of time-to-treatment. Second, individuals with longer time-to-treatment or with persistent symptoms may have been more likely to respond to the questionnaire, potentially resulting in an overestimation of time-to-treatment and its association with PTLDS. To mitigate participation bias, we employed inverse probability weighting. Third, the study population was diagnosed with Lyme disease at Geisinger, a single integrated health system. However, Geisinger has more than 44 community practice sites, 12 hospital campuses, and more than 20 urgent care clinics across a large geographic region; thus, the findings reflect the practices of Lyme disease diagnosis across a range of clinical settings. Moreover, questionnaires captured information on experiences within and outside of Geisinger. Finally, our findings may be subject to same-source bias due to the use of self-reported data for both exposures and outcomes.

## Conclusions

In a population-based study of Lyme disease in Pennsylvania, treatment delays, defined as time-to-treatment >30 days, were reported by nearly one-third of individuals with Lyme disease. Delays before and after contacting a medical professional had common and unique risk factors. Delayed treatment was associated with PTLDS. To improve long-term outcomes of Lyme disease, strategies for preventing delayed treatment should aim to reduce both the time before and after contacting a medical professional.

## Data Availability Statement

The raw data supporting the conclusions of this article will be made available by the authors, without undue reservation.

## Ethics Statement

The studies involving human participants were reviewed and approved by Geisinger Institutional Review Board. Written informed consent for participation was not required for this study in accordance with the national legislation and the institutional requirements.

## Author Contributions

AH, MP, and BS conceived and designed the analysis. CN performed the data analysis. AH, BS, MP, KM, AR, JA, CH, and BS contributed to the interpretation of results. All authors contributed to the writing and final review of the manuscript.

## Conflict of Interest

The authors declare that the research was conducted in the absence of any commercial or financial relationships that could be construed as a potential conflict of interest.
