# Supplementary material for: Risk Factors and Outcomes of Treatment Delays in Lyme Disease: A Population-Based Retrospective Cohort Study
Source: Front Med (Lausanne). 2020 Nov 26;7:560018. doi: 10.3389/fmed.2020.560018 (PMC7726265; doi:10.3389/fmed.2020.560018)
Supplement: Supplementary file 1 [file Data_Sheet_1.DOCX]

| **Table S1. Evaluation of goodness-of-fit of unweighted and weighted logistic regression models** | | |
| --- | --- | --- |
| **Logistic regression models** | **Unweighted model**  **Hosmer-Lemeshow chi-square (p-value)** | **Weighted model^1^**  **F-statistic (p-value)^2^** |
| Logistic regression analysis of factors related to delays in contacting a medical professional for Lyme disease | 1.25 (0.9961) | 0.65 (0.7560) |
| Logistic regression analysis of factors related to delays between healthcare contact and treatment for Lyme disease | 7.32 (0.5025) | 0.44 (0.9159) |
| ^1^Weighted by participation rates, ^2^F-statistic used to evaluate goodness-of-fit for models of complex survey data | | |

**Figure S1. Standardized residuals versus predicted probability of delay in contacting a medical professional (study sample, unweighted)**


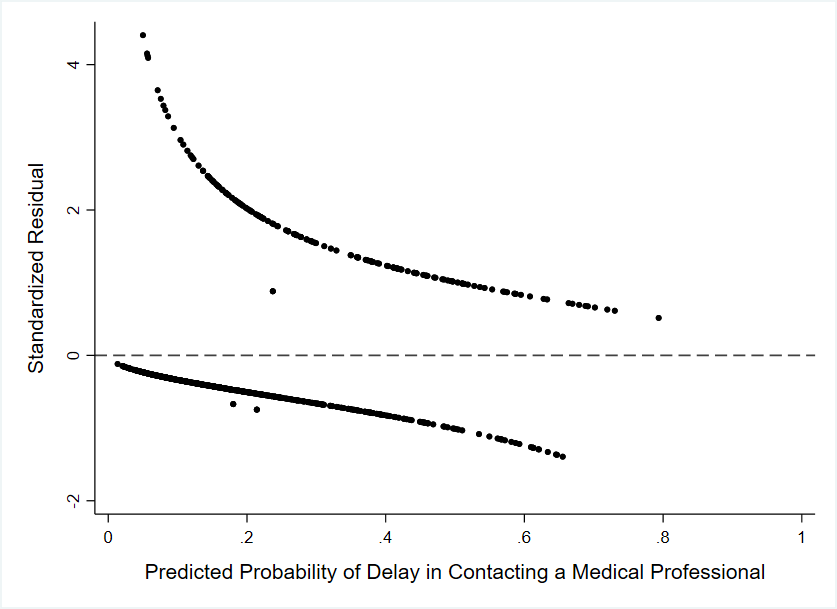


**Figure S2. Standardized residuals versus predicted probability of delay in contacting a medical professional (source population, weighted for participation rates)**


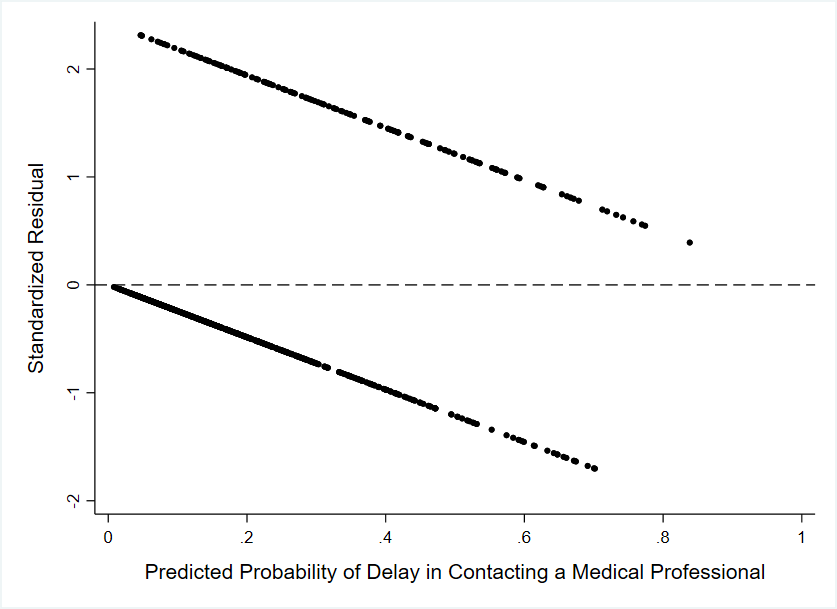


**Figure S3. Standardized residuals versus predicted probability of delay between healthcare contact and treatment (study sample, unweighted)**


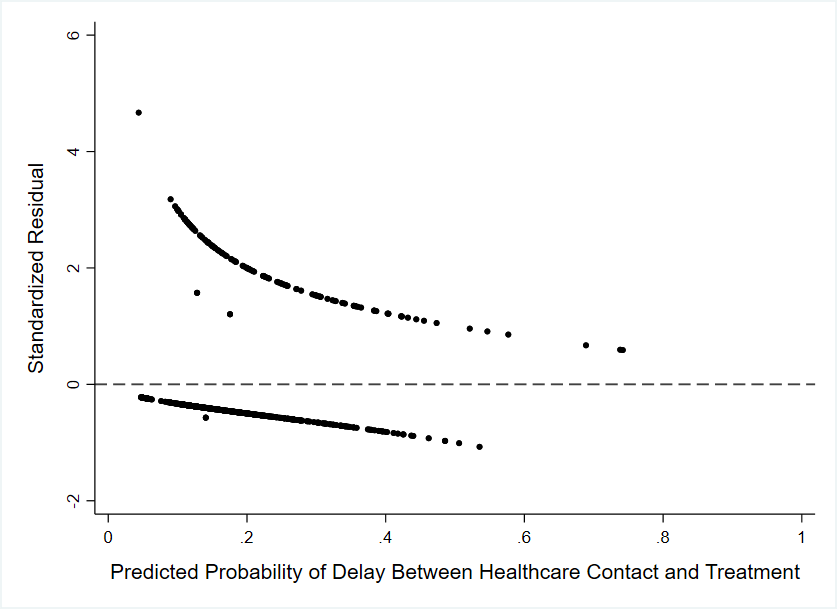


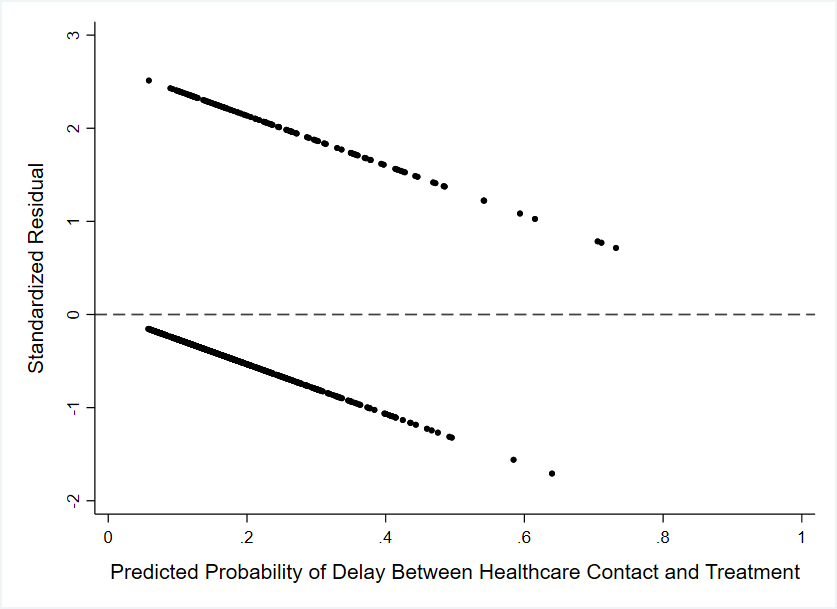


**Figure S4. Standardized residuals versus predicted probability of delay between healthcare contact and treatment (source population, weighted for participation rates)**
